# Supplementary material for: A nucleotide-sensing oligomerization mechanism that controls NrdR-dependent transcription of ribonucleotide reductases
Source: Nat Commun. 2022 May 16;13:2700. doi: 10.1038/s41467-022-30328-1 (PMC9110341; doi:10.1038/s41467-022-30328-1)
Supplement: Supplementary file 1 — Supplementary Information [file 41467_2022_30328_MOESM1_ESM.pdf]

Supplementary information

A nucleotide-sensing oligomerization mechanism that controls NrdR-dependent transcription of ribonucleotide reductases

Inna Rozman Grinberg, Markel Martínez-Carranza, Ornella Bimai, Ghada Nouaïria, Saher Shahid, Daniel Lundin, Derek T. Logan*, Britt-Marie Sjöberg* and Pål Stenmark*

Supplementary Figure 1-14

Supplementary Table 1

Supplementary Table 2

Supplementary Figure 1

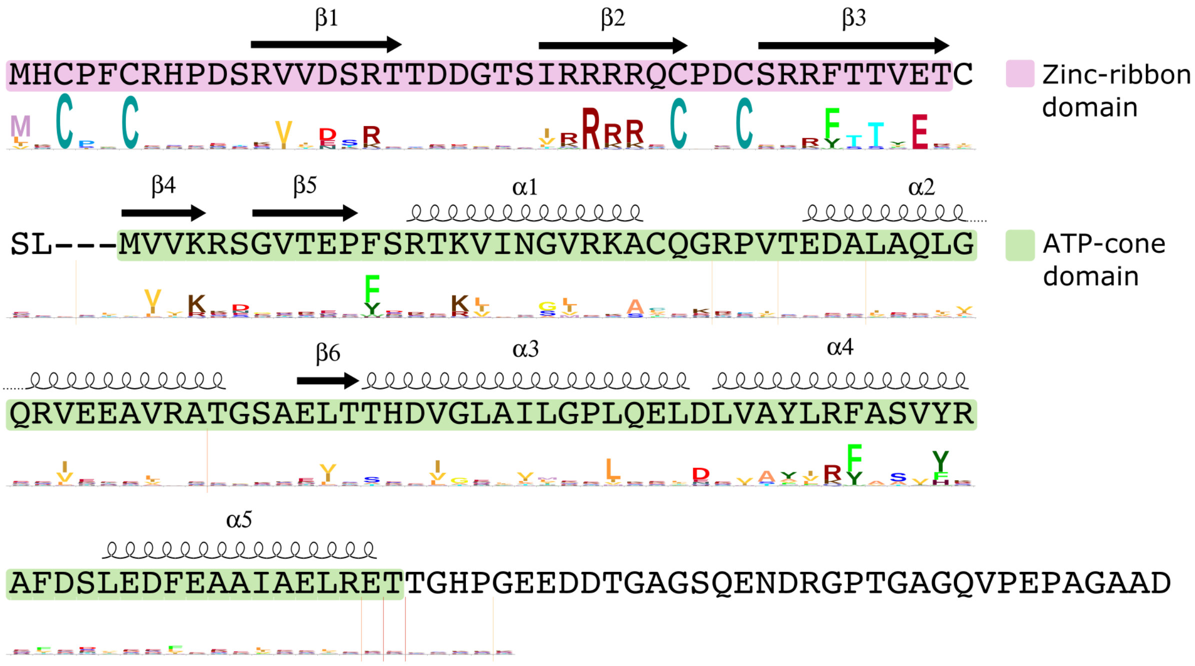

Supplementary Figure 1. *S. coelicolor* NrdR (UNIPROT ID O69980) sequence with secondary structure annotations, based on the dATP/ATP-loaded tetrameric NrdR structure bound to its cognate DNA (PDB ID 7P3F). The zinc-ribbon domain (residues 1-43) is highlighted in pink and the ATP-cone domain (residues 47-147) is highlighted in green. The logo was created by Skylign[1] as described in Methods.

Supplementary Figure 2

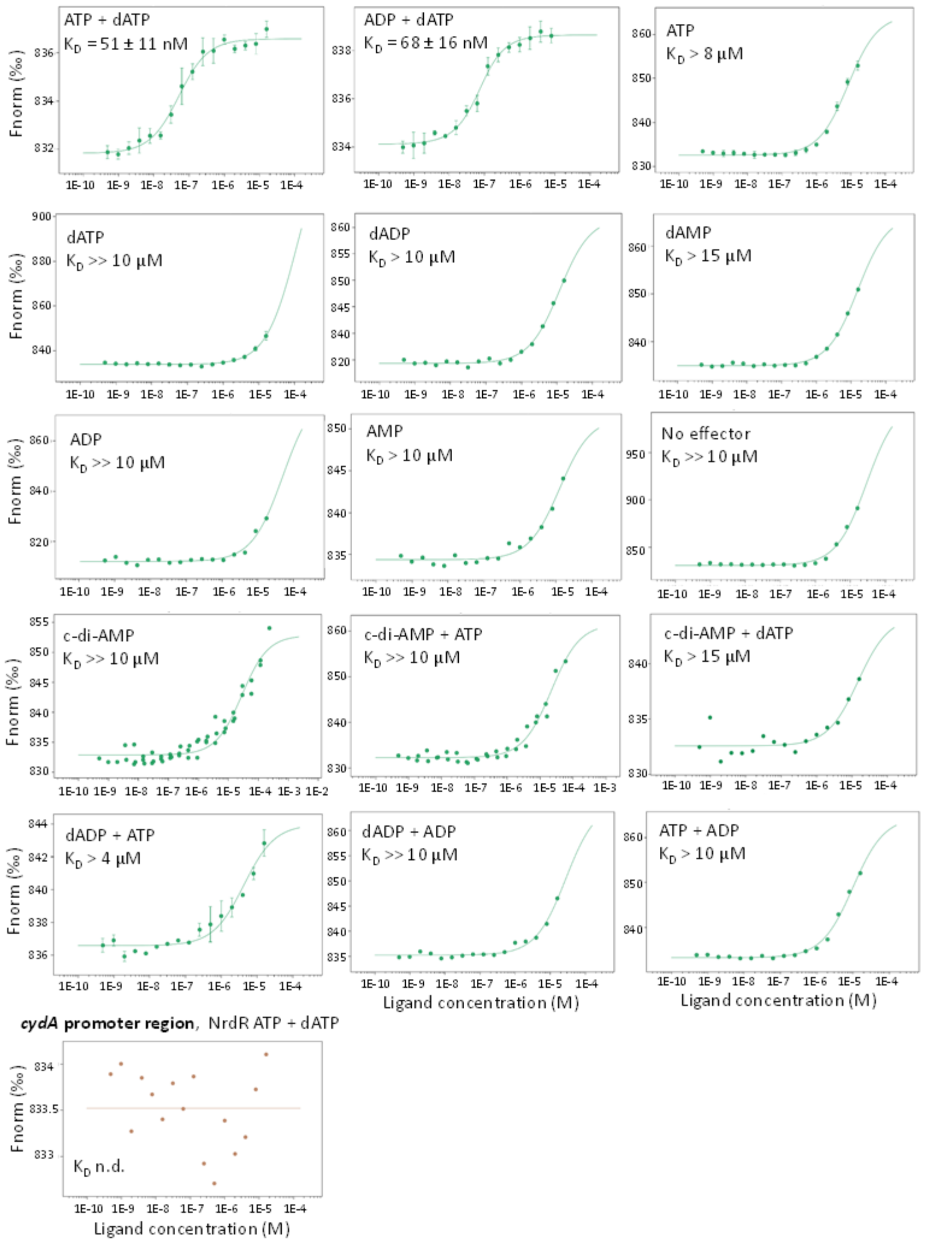

Supplementary Figure 2. Binding of nucleotide-loaded *S. coelicolor* NrdR to NrdR boxes in *nrdRJ* promoter and to the *cydA* promoter region (negative control, lower left panel), determined by MST. Plots of the normalized fluorescence F_{norm} (‰) from *T*-Jump and Thermophoresis vs. the concentration of ligand (NrdR) are shown. Lines represent fits of the data points using the K_D fit derived from the law of mass action. For single nucleotides and their combinations other than dATP + ATP and dATP + ADP, the fits resulted in K_Ds in the range of 4 - 50 μM (in most cases higher than 10 μM). The actual K_D cannot be determined, since the curves do not reach a plateau. Since these fitted K_Ds reflect binding affinities approximately 100 - 1000 times lower than those of dATP + ATP and dATP + ADP loaded NrdR (K_Ds of 51 ± 11 and 68 ± 16 nM respectively), we believe that they are not physiologically relevant and reflect non-specific binding. K_Ds between 10 μM and 19 μM are denoted as K_D>10 μM, and above 19 μM as K_D >>10μM. n.d. not determined. K_D and standard deviations (mean values ± SD) were calculated using fits from at least three individual titrations.

Supplementary Figure 3

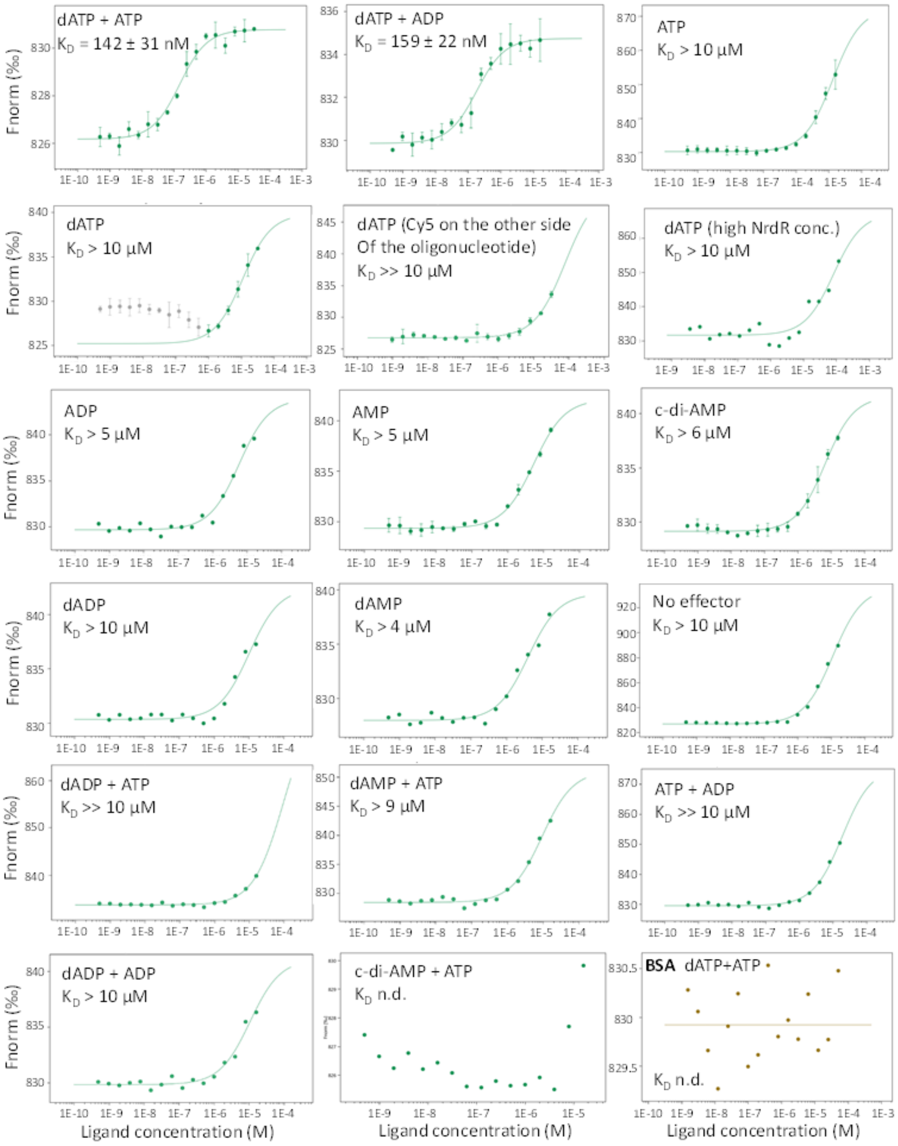

Supplementary Figure 3. Binding of nucleotide-loaded *S. coelicolor* NrdR and BSA (negative control, lower right panel) to NrdR-boxes in the *nrdAB* promoter region determined by MST. Plots of the normalized fluorescence F_{norm} (‰) from *T*-Jump and Thermophoresis vs. the concentration of ligand (NrdR, BSA) are shown. Lines represent fits of the data points using the K_D fit derived from the law of mass action. For single nucleotides and their combinations other than dATP + ATP and dATP + ADP, the fits resulted in K_Ds in the range of 4 - 50 µM (in most cases higher than 10 µM). The actual K_D cannot be determined, since the curves do not reach a plateau, even when using higher (117 µM) NrdR concentrations in the assay (such a titration for NrdR + dATP is shown). Since these fitted K_Ds reflect binding affinities at least 25 times lower than those of dATP + ATP and dATP + ADP loaded NrdR (K_Ds of 142 ± 31 and 159 ± 22 nM respectively), we believe that they are not physiologically relevant and reflect non-specific binding. K_Ds between 10 µM and 19 µM are denoted as K_D>10 µM, and above 19 µM as K_D >>10µM. For titration with dATP-loaded NrdR, a biphasic curve was detected, which disappeared when the Cy5 position was changed to the opposite side of the oligonucleotide, close to the 2nd NrdR box. n.d. not determined. K_D and standard deviations (mean values ± SD) were calculated using fits from at least three individual titrations.

Supplementary Figure 4

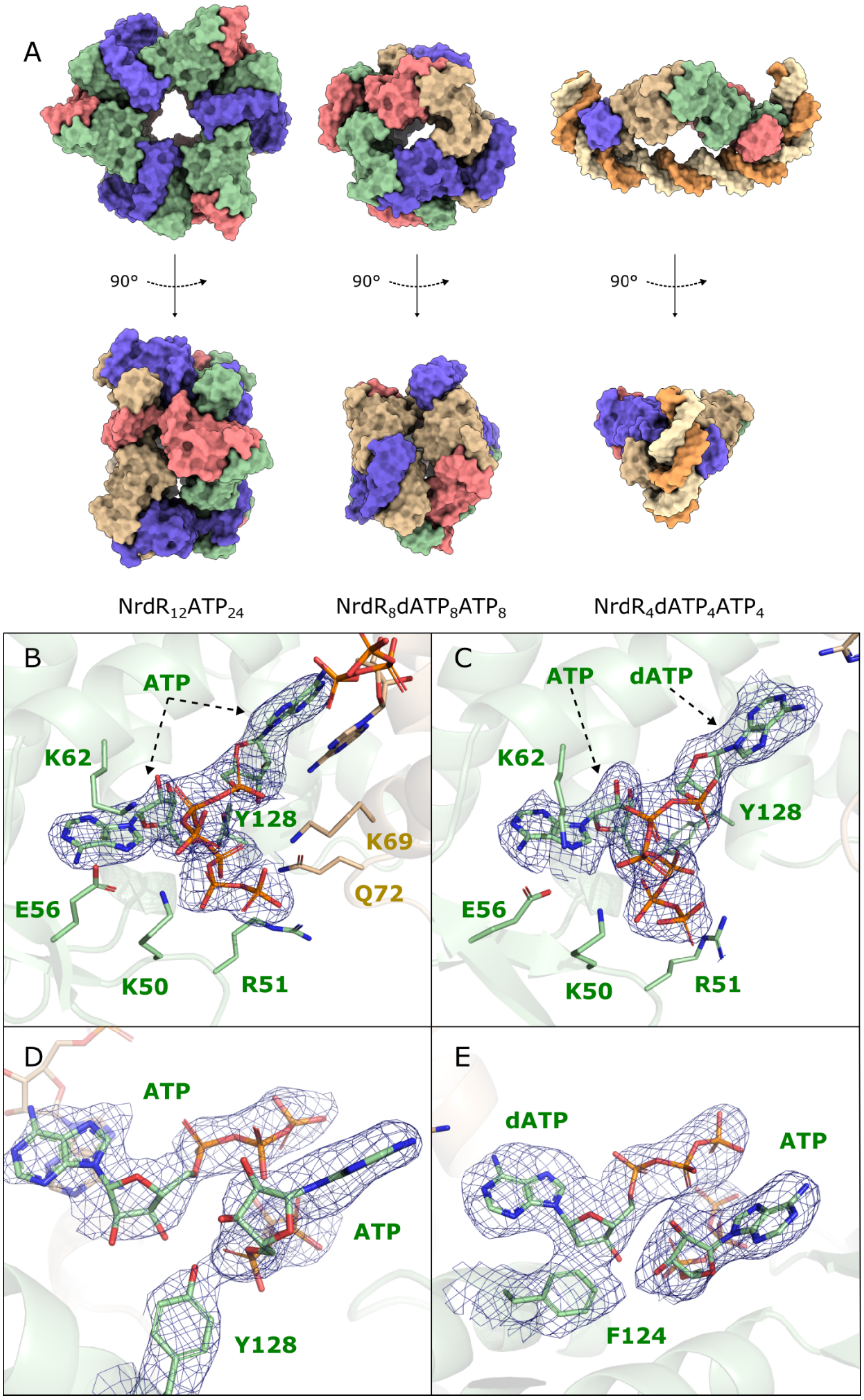

Supplementary Figure 4. (**A**) Surface representation of NrdR cryo-EM models: ATP-loaded NrdR dodecamer (left), dATP/ATP-loaded NrdR octamer (center), dATP/ATP-loaded NrdR tetramer bound to DNA oligomer containing two NrdR boxes (right). NrdR monomers in each tetramer assembly are colored yellow, blue, green and red. Forward strand is colored orange, the reverse strand is colored light yellow. (**B, D**) Nucleotide coordination in the ATP-loaded dodecameric structure, chain B is colored in green and chain A is colored in beige. (**C, E**) Nucleotide coordination in the dATP/ATP-loaded NrdR tetrameric structure, chain B is colored in green.

Supplementary Figure 5

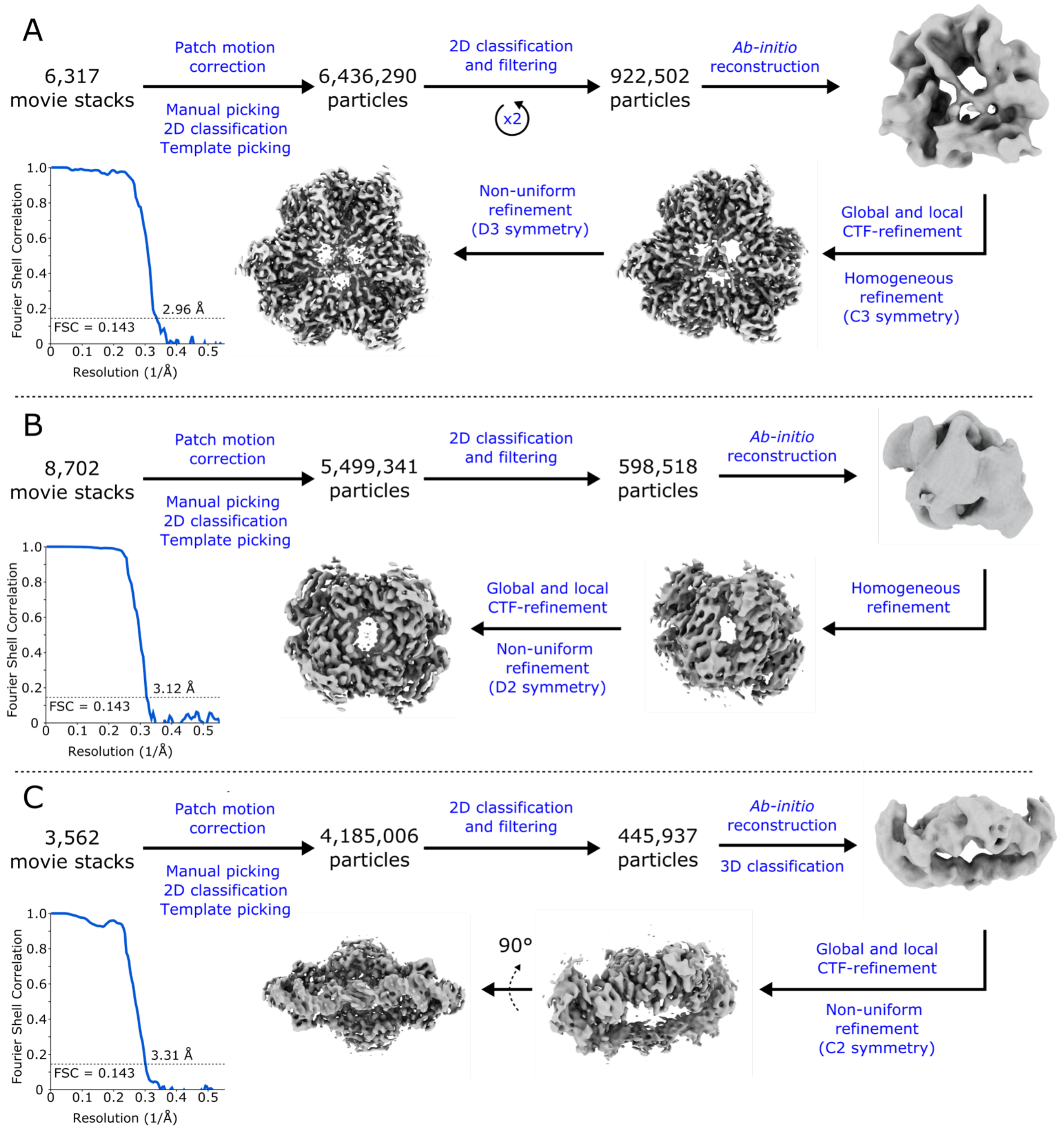

Supplementary Figure 5. Cryo-EM processing summary of the NrdR dodecameric (**A**), octameric (**B**) and tetrameric (**C**) structures.

Supplementary Figure 6

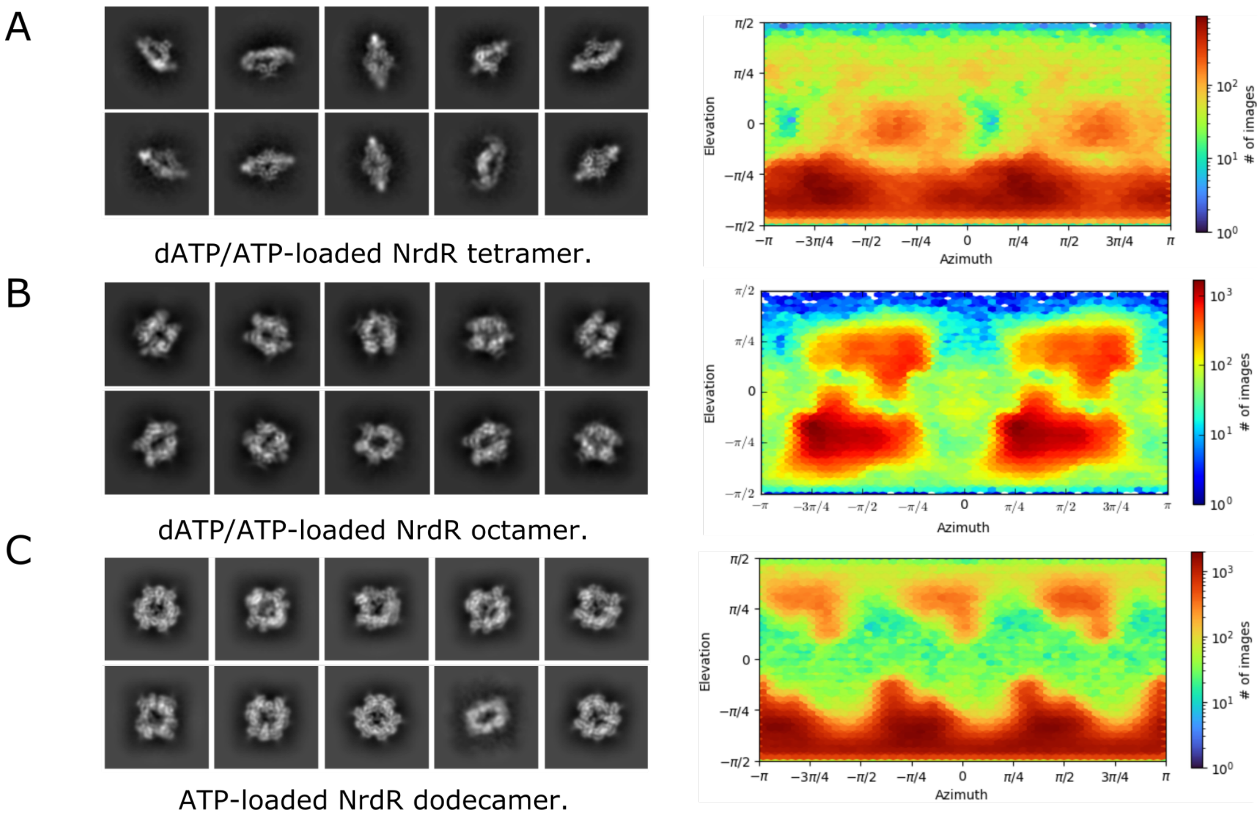

Supplementary Figure 6. 2D classification results (left) and particle orientation distribution (right) for NrdR tetrameric (**A**), octameric (**B**) and dodecameric (**C**) structures.

Supplementary Figure 7

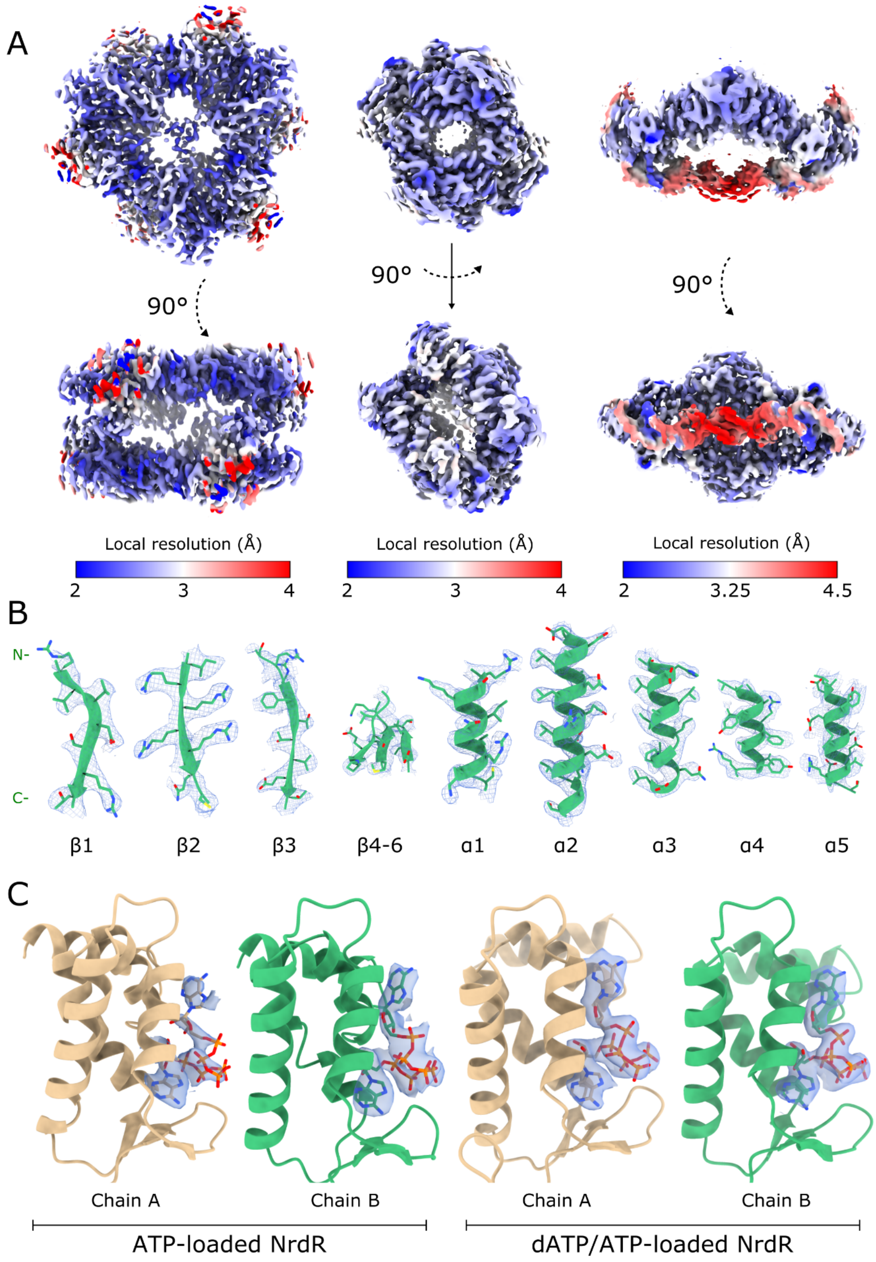

Supplementary Figure 7. Agreement between map and model. (**A**) Local resolution maps of the ATP-loaded NrdR dodecamer (left), dATP/ATP-loaded NrdR octamer (center) and dATP/ATP-loaded NrdR tetramer bound to its cognate DNA (right). The local resolution maps were calculated in cryoSPARC[2] and displayed and captured in ChimeraX[3]. (**B**) Secondary structure elements of the NrdR model and ATP-loaded cryo-EM map (chain A). The map was displayed at a threshold of 0.4, and 0.22 for helix 5. (**C**) ATP-cone domain model and ATP-loaded NrdR map displayed at a threshold of 0.37 for chains A and B (left), and at a threshold of 0.4 for the dATP/ATP-loaded NrdR map (right).

Supplementary Figure 8

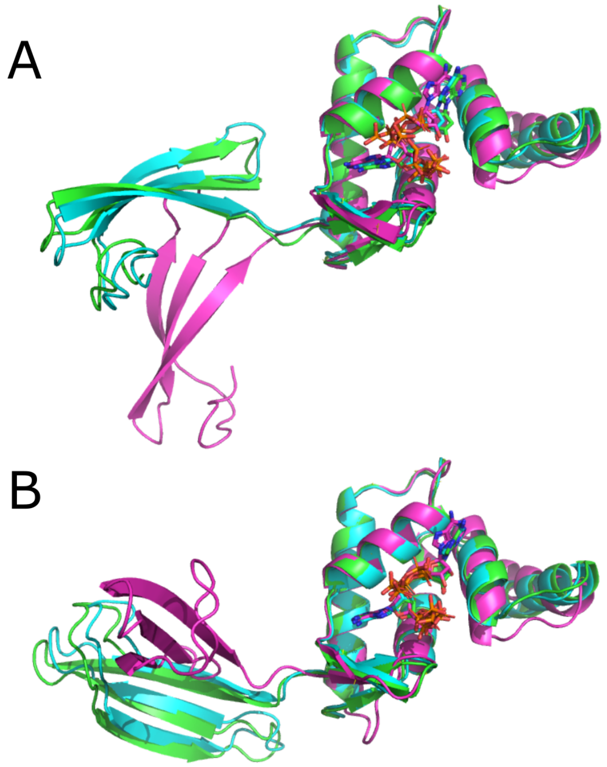

Supplementary Figure 8. Superposition of NrdR monomers from the tetrameric (green), octameric (cyan) and dodecameric (magenta) structures. All monomers were aligned using only the ATP-cone domain. (**A**) Overlay of the A chains from the three NrdR structures. (**B**) Overlay of the B chains of the three NrdR structures.

Supplementary Figure 9

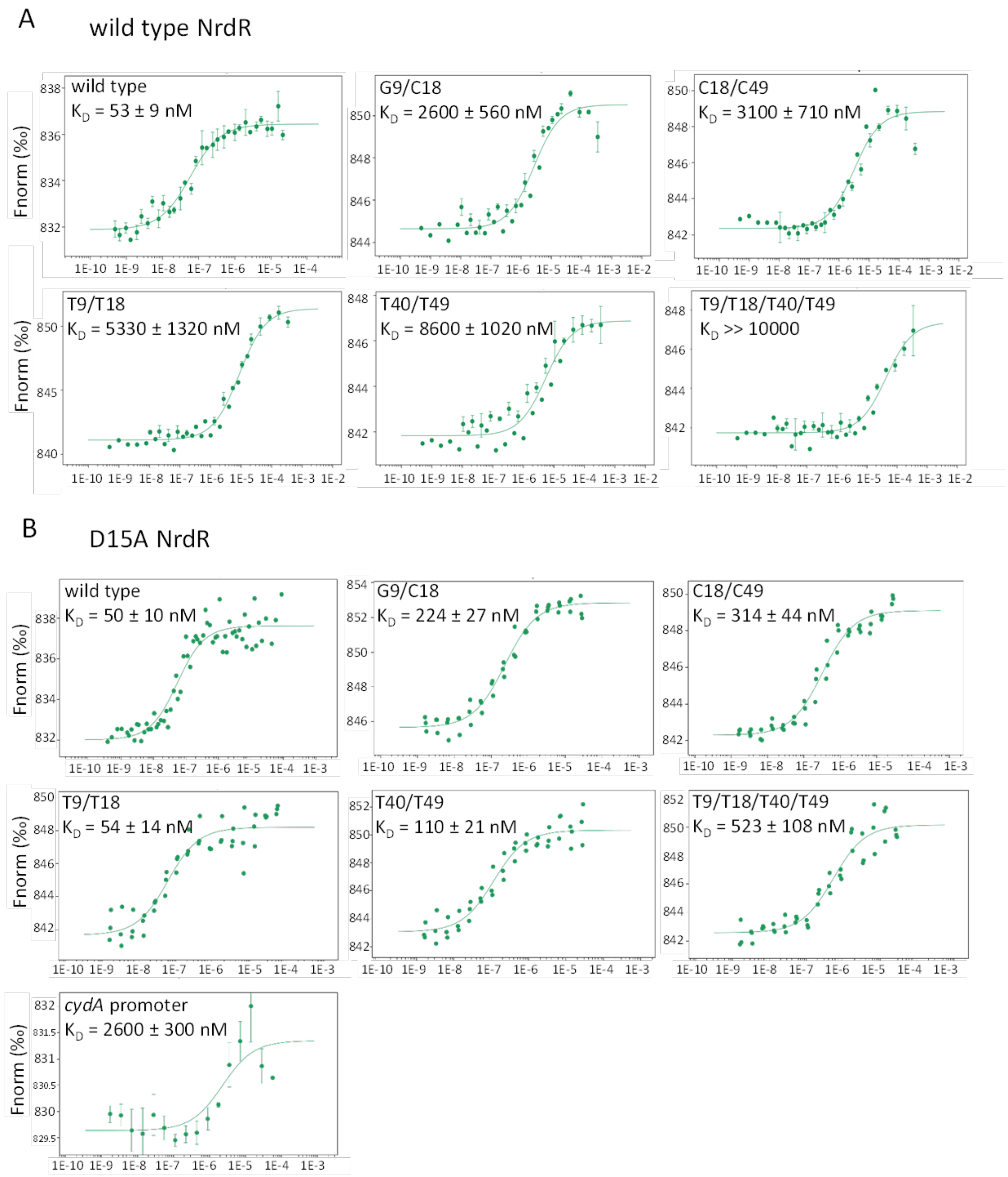

Supplementary Figure 9. Binding of wild type **(A)** and D15A **(B)** mutant *S. coelicolor* NrdR proteins in the presence of 1 mM ATP and 1 mM dATP to wild type and mutated NrdR-boxes in the *nrdRJ* promoter region and to *cydA* promoter region determined by MST. Plots of the normalized fluorescence *F*norm (‰) from *T*-Jump and Thermophoresis vs. the concentration of ligand (NrdR) are shown. Lines represent fits of the data points using the K_D fit derived from the law of mass action. A binding constant of >>10 µM indicates a K_D above 19 µM; n.d. not determined. K_D and standard deviations (mean values ± SD) were calculated using fits from at least three individual titrations.

Supplementary Figure 10

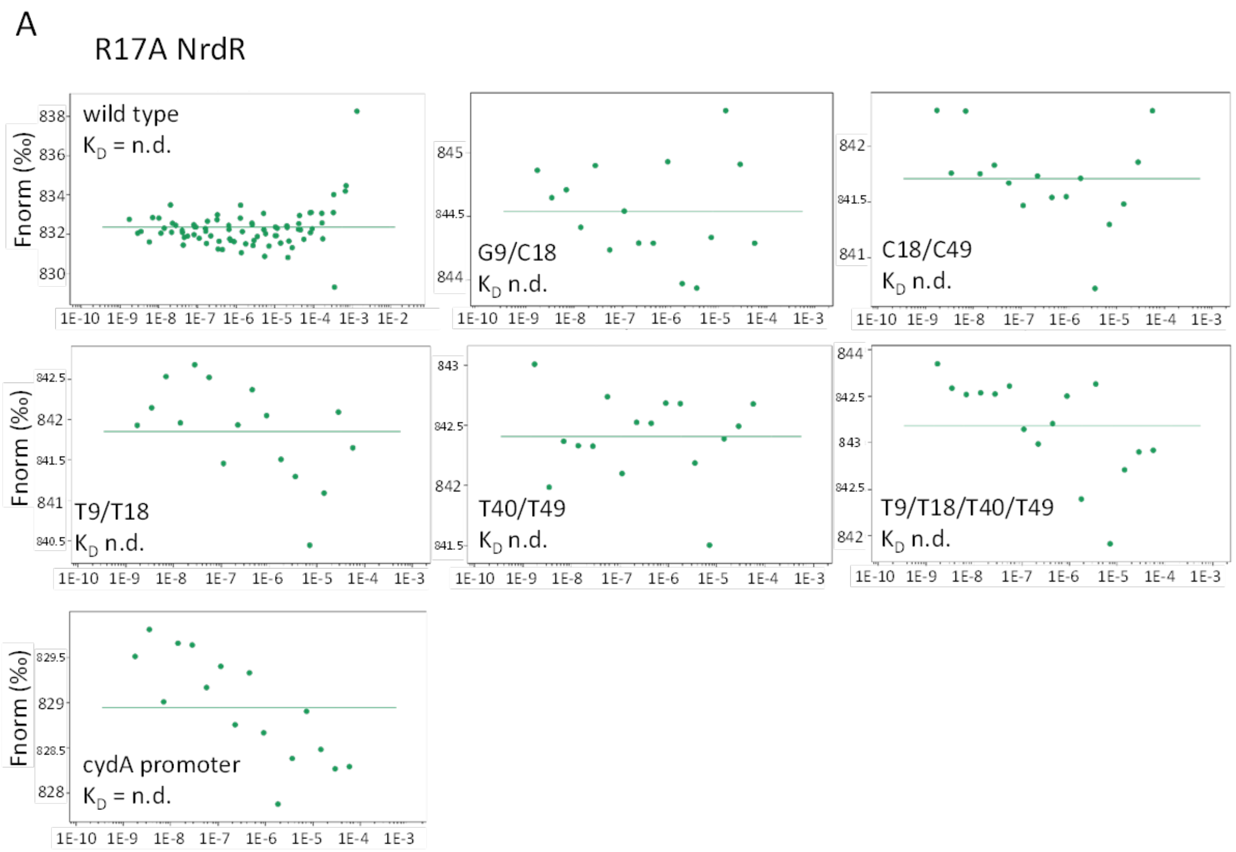

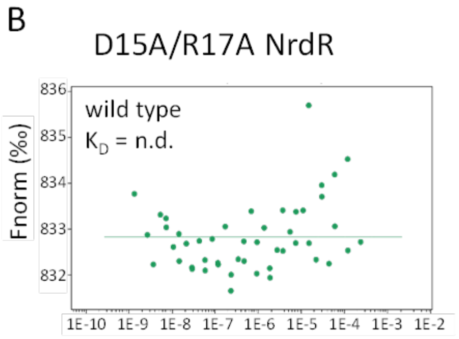

Supplementary Figure 10. Binding of R17A (**A**) and D15A/R17A (**B**) mutant *S. coelicolor* NrdR proteins in the presence of 1 mM ATP and 1 mM dATP to wild type and mutated NrdR-boxes in the *nrdRJ* promoter region and to *cydA* promoter region determined by MST. Plots of the normalized fluorescence *F*norm (‰) from *T*-Jump and Thermophoresis vs. the concentration of ligand (NrdR) are shown. n.d. not determined.

Supplementary Figure 11

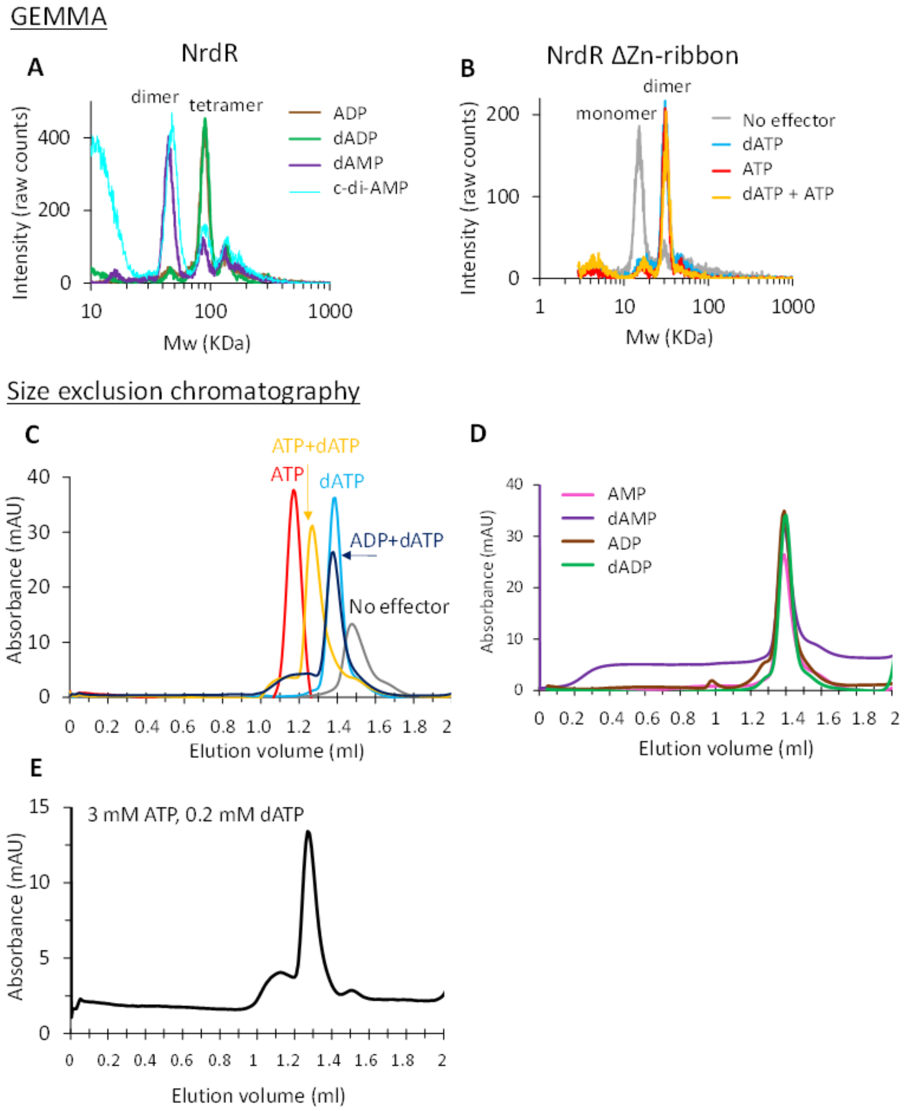

Supplementary Figure 11. Oligomeric state analysis of NrdR. Gas-phase electrophoretic mobility molecular analyses (GEMMA) of nucleotide-loaded *S. coelicolor* NrdR (**A**) and ATP-cone only mutant NrdRΔNΔC (ΔZn-ribbon, ΔC-term NrdR) (**B**). Size exclusion chromatography of *S. coelicolor* NrdR in the presence of different nucleotides (**C, D**) and in a combination of ATP and dATP at physiological concentrations (**E**). Red, ATP; light blue, dATP; orange, dATP + ATP; dark blue, dATP + ADP; brown, ADP; green, dADP; magenta, AMP; purple, dAMP; cyan, c-di-AMP; grey, no effector. When used to estimate molecular size, SEC assumes globular proteins, and may give too high values for proteins of other shapes, and molecular size may only be qualitative. In a first set of experiments we found that dATP-loaded NrdR eluted slower than nucleotide-free NrdR and had approximately twofold higher calculated molecular weight. Assuming apo-NrdR being a dimer, this is consistent with dATP inducing a tetramer (C). Most other adenosine nucleotides also induced the same complex, as did dATP, whereas addition of ATP induced a very large oligomeric complex (C, D). Simultaneous addition of dATP and ATP (C, E) induced an oligomeric complex that eluted between the dATP- and the ATP-induced complexes.

Supplementary Figure 12

A

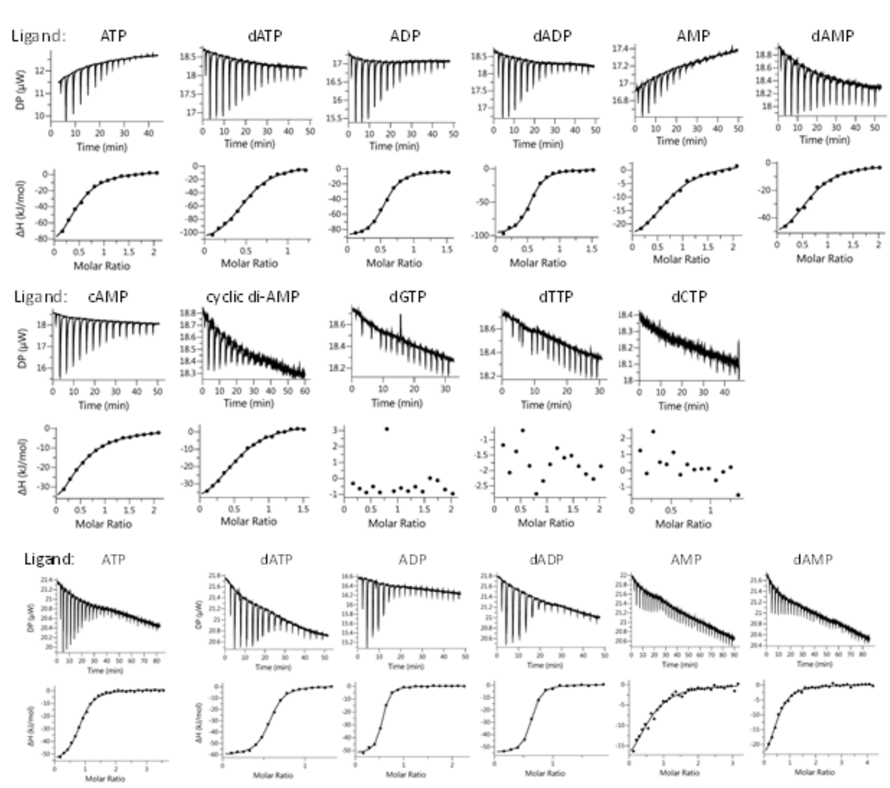

C

Ligand	N at 20°C (at 10°C)	K_d (µM) at 20°C (at 10°C)	ΔH (kJ/mol) at 20°C (at 10°C)	ΔG (kJ/mol) at 20°C (at 10°C)	-TΔS (kJ/mol) at 20°C (at 10°C)
ATP	0.51 ± 0.08 (0.83)	3.95 ± 0.5 (1.25)	-112 ± 6 (-57)	-30 (-32)	82 ± 6 (25)
dATP	0.47 ± 0.01 (0.55)	1.09 ± 0.22 (0.269)	-108 ± 8 (-62)	-34 (-36)	75 ± 8 (26)
ADP	0.52 ± 0.02 (0.5)	0.602 ± 0.062 (0.225)	-81 ± 13 (-53)	-35 (-36)	46 ± 13 (17)
dADP	0.46 ± 0.06 (0.58)	0.429 ± 0.047 (0.213)	-91 ± 6 (-55)	-36 (-36)	56 ± 6 (19)
AMP	0.8 ± 0.07 (0.59)	3.83 ± 0.96 (5.85)	-32 ± 5 (-25)	-31 (-28)	2 ± 6 (-4)
dAMP	0.59 ± 0.04 (0.48)	3.84 ± 0.33 (3.76)	-60 ± 0.6 (-32)	-30 (-29)	30 ± 0.6 (2)
cAMP	0.51 ± 0.13	35 ± 4	-54 ± 15	-25	29 ± 15
c-di-AMP	0.46 ± 0.06	2.72 ± 0.74	-48 ± 5	-31	17 ± 5
dTTP, dGTP, dCTP	n.d.	n.d.	n.d.	n.d.	n.d.

Supplementary Figure 12. ITC analyses of nucleotide binding to *S. coelicolor* NrdR. Representative ITC thermograms obtained by titration of specified ligands to NrdR at 20 °C (**A**) and 10 °C (**B**). Isothermal calorimetric enthalpy changes (upper panels) and resulting binding isotherms (lower panels) are shown. (**C**) Thermodynamic parameters of ligand binding to NrdR at 20 °C and 10 °C (in parenthesis). Binding isotherms were fitted using a one-set-of-sites binding model. Values for titration at 20 °C are reported as the mean ±SD of three titrations. Values for titration at 10 °C are based on a single binding experiment for each ligand. All titrations were performed as described in Methods. n.d. = not detected.

Supplementary Figure 13

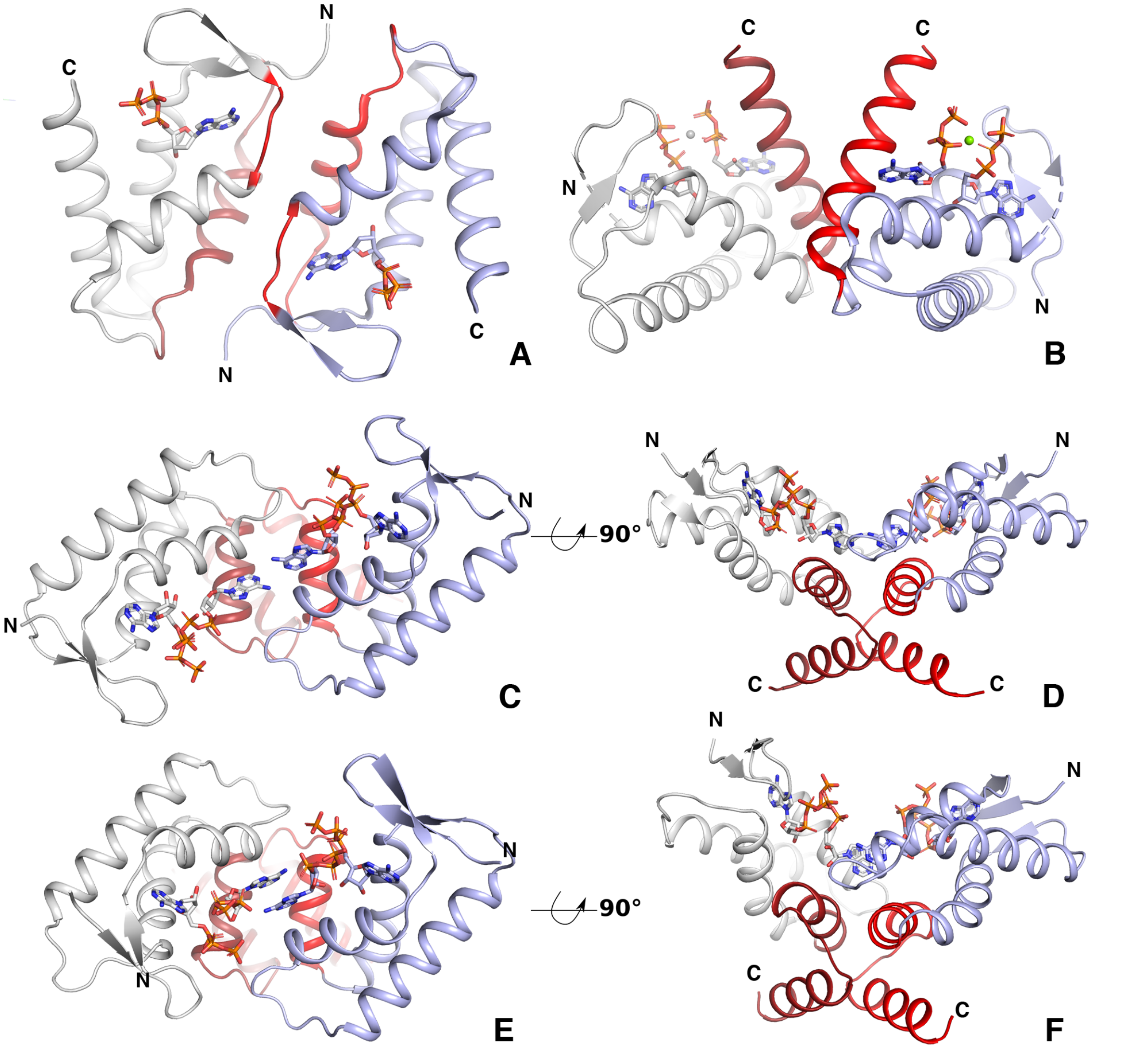

Su

Supplementary Figure 13. Differences in structure and homotypic molecular interactions of three types of ATP-cones. In all panels the interacting ATP-cones are coloured in light gray and pale blue respectively. The main secondary structural elements involved in intermolecular interactions are highlighted in firebrick and red for the left- and right-hand monomers respectively. Nucleotides are shown in stick representation. (**A**) ATP-cone dimer from the inactive dATP-induced hexamer of human RNR (PDB ID 6AUI). (**B**) ATP-cone dimer from the dATP-induced tetramer of *L. blandensis* NrdB (5OLK). (**C**) ATP-cone dimer from the octameric form of *S. coelicolor* NrdR loaded with dATP and ATP. (**D**) Alternative view rotated by 90° around a horizontal axis relative to panel C. (**E**) ATP-cone dimer from the dodecameric ATP-loaded form of *S. coelicolor* NrdR. The right-hand monomer of the dimer has been superimposed on that of the octameric form using residues 47-117, i.e. excluding the two C-terminal helices. The RMS deviation in Cα positions for 71 atoms is 0.60 Å. (**F**) Alternative view rotated by 90° around a horizontal axis relative to panel E.

Supplementary Figure 14

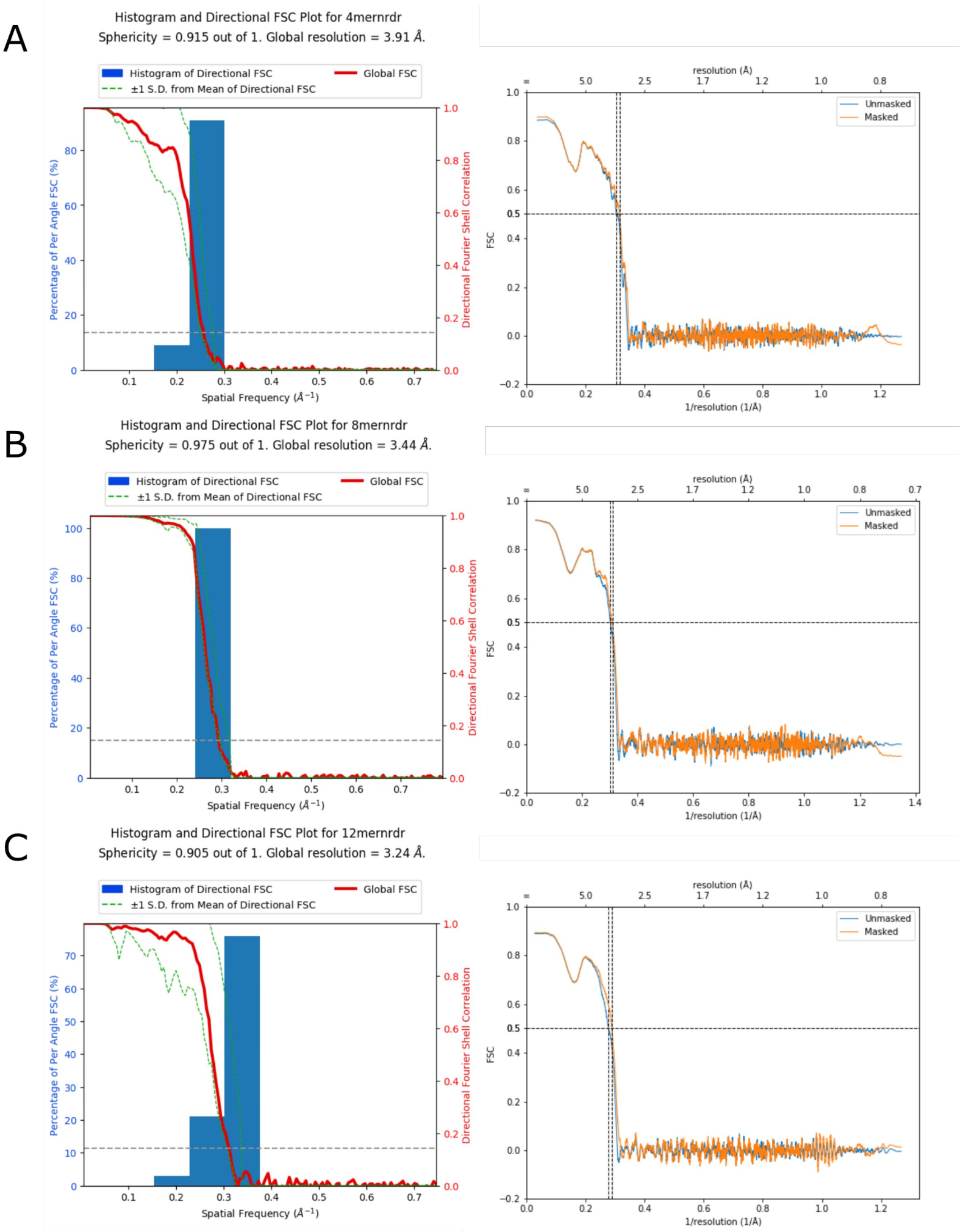

Supplementary Figure 14. 3DFSC analysis[4] of NrdR tetrameric (**A**), octameric (**B**) and dodecameric (**C**) structures (left), and their respective model-to-map FSC graphs generated in Phenix (version 1.19.2-4158-000)[5].

Supplementary Table 1. Data collection, refinement and validation statistics.

	ATP-loaded NrdR dodecamer (EMDB-13178) PDB 7P37)	dATP/ATP-loaded NrdR octamer (EMDB-13182) PDB 7P3Q)	dATP/ATP-loaded NrdR tetramer bound to DNA (EMDB-13179) PDB 7P3F)
**Data collection and processing**			
Magnification	120.000	215.000	120.000
Voltage (kV)	300	300	300
Electron exposure (e-/A^2)	50.0	60.2	50.0
Defocus range (µm)	-1.0 – -2.5	-1.4 – -3.2	-1.4 – -3.2
Pixel size (Å)	0.67	0.63	0.67
Symmetry imposed	D3	D2	C2
Initial particle images	1206376	934270	1168345
Final particle images	922502	598518	445937
Map resolution (Å) (FSC = 0.143)	2.96	3.12	3.31
**Refinement**			
Initial model used (PDB)	-	7P37	7P37
Map sharpening *B* factor (Å^2)	172.2	189.5	194.9
Model composition			
Non-hydrogen atoms	14496	9632	6878
Protein residues	1764	1176	588
DNA residues	-	-	100
Ligands	36	24	12
B-factors (min/max/mean, Å^2)			
Protein	11.73/54.01/30.44	18.66/113.83/55.78	22.17/80.99/48.31
DNA	-	-	20.00/175.46/141.52
Ligands	26.84/69.44/34.15	22.95/132.98/31.78	20.00/123.58/44.26
R.M.S. deviations			
Bond lengths (Å)	0.006	0.009	0.009
Bond angles (°)	1.077	1.510	1.413
**Validation**			
MolProbity score	1.48	1.88	1.85
Clashscore	7.21	11.26	7.27
Poor rotamers (%)	0	0.4	1.59
Ramachandran plot			
Overall Z-score [RMSD]	1.79	0.44	-1.22
Favored (%)	97.59	95.52	95.69
Allowed (%)	2.41	4.48	4.31
Disallowed (%)	0	0	0

Supplementary Table 2. Oligonucleotides used.

Gene	Name	Nucleotide sequence
nrdAB	nrdAB_Cy5_sense	5'-Cy5-CCGGGACACAACATCTGGGGGTGCTCGCGTCCCCCGGCACAAGATGTATGCTCATGC-3'
	nrdAB_antisense	5'-GCATGAGCATACATCTTGTGCCGGGGGACGCGAGCACCCCCAGATGTTGTGTCCCGG-3'
	nrdAB_ sense	5'-CCGGGACACAACATCTGGGGGTGCTCGCGTCCCCCGGCACAAGATGTATGCTCATGC-3'
	nrdAB_Cy5_antisense	5'-Cy5-GCATGAGCATACATCTTGTGCCGGGGGACGCGAGCACCCCCAGATGTTGTGTCCCGG-3'
nrdRJ	nrdRJ_Cy5_sense	5'-Cy5-GCCAATCCCCACATCTAGTGGTTGGATAGCGTGAGCAGCCCACAAGTTGTGGTCCCC-3'
	nrdRJ_antisense	5'-GGGGACCACAACTTGTGGGCTGCTCACGCTATCCAACCACTAGATGTGGGGATTGGC-3'
	G9/C18_Cy5-sense	5'-Cy5-GCCAATCCGCACATCTACTGGTTGGATAGCGTGAGCAGCCCACAAGTTGTGGTCCCC-3
	G9/C18_antisense	5'-GGGGACCACAACTTGTGGGCTGCTCACGCTATCCAACCAGTAGATGTGCGGATTGGC-3'
	C18/C49_Cy5-sense	5'-Cy5-GCCAATCCCCACATCTACTGGTTGGATAGCGTGAGCAGCCCACAAGTTCTGGTCCCC-3'
	C18/C49_antisense	5'-GGGGACCAGAACTTGTGGGCTGCTCACGCTATCCAACCAGTAGATGTGGGGATTGGC-3'
	T9/T18_Cy5-sense	5'-Cy5-GCCAATCCCCACATCTAGTGGTTGGATAGCGTGAGCAGCTCACAAGTTTTGGTCCCC-3'
	T9/T18_antisense	5'-GGGGACCAAAACTTGTGAGCTGCTCACGCTATCCAACCACTAGATGTGGGGATTGGC-3'
	T40/T49_Cy5_sense	5'-Cy5-GCCAATCCTCACATCTATTGGTTGGATAGCGTGAGCAGCCCACAAGTTGTGGTCCCC-3'
	T40/T49_antisense	5'-GGGGACCACAACTTGTGGGCTGCTCACGCTATCCAACCAATAGATGTGAGGATTGGC-3'
	T9/T18/T40/T49_Cy5_sense	5'-Cy5-GCCAATCCTCACATCTATTGGTTGGATAGCGTGAGCAGCTCACAAGTTTTGGTCCCC-3'
	T9/T18/T40/T49_antisense	5'-GGGGACCAAAACTTGTGAGCTGCTCACGCTATCCAACCAATAGATGTGAGGATTGGC-3'
	cryoEM_sense	5'-GCCAATCCCCACATCTAGTGGTTGGATAGCGTGAGCAGCCCACAAGTTGTGGTCCCC-3'
	cryoEM_antisense	5'-GGGGACCACAACTTGTGGGCTGCTCACGCTATCCAACCACTAGATGTGGGGATTGGC-3'
cydA	cydA_Cy5_sense	5'-Cy5-GTAGGTCATAATTGCTTGTGAATGTGAACGCGTTCACAAGCGTGTCCCGATTGCGCC-3'
	cydA_antisense	5'-GGCGCAATCGGGACACGCTTGTGAACGCGTTCACATTCACAAGCAATTATGACCTAC-3

Supplementary References

1 Wheeler, T. J., Clements, J. & Finn, R. D. Skylign: a tool for creating informative, interactive logos representing sequence alignments and profile hidden Markov models. *BMC Bioinformatics* **15**, 7, doi:10.1186/1471-2105-15-7 (2014).

2 Punjani, A., Rubinstein, J. L., Fleet, D. J. & Brubaker, M. A. cryoSPARC: algorithms for rapid unsupervised cryo-EM structure determination. *Nat Methods* **14**, 290-296, doi:10.1038/nmeth.4169 (2017).

3 Pettersen, E. F. *et al.* UCSF ChimeraX: Structure visualization for researchers, educators, and developers. *Protein Sci* **30**, 70-82, doi:10.1002/pro.3943 (2021).

4 Tan, Y. Z. *et al.* Addressing preferred specimen orientation in single-particle cryo-EM through tilting. *Nat Methods* **14**, 793-796, doi:10.1038/nmeth.4347 (2017).

5 Liebschner, D. *et al.* Macromolecular structure determination using X-rays, neutrons and electrons: recent developments in Phenix. *Acta Crystallogr D Struct Biol* **75**, 861-877, doi:10.1107/S2059798319011471 (2019).
