# Supplementary material for: A nucleotide-sensing oligomerization mechanism that controls NrdR-dependent transcription of ribonucleotide reductases
Source: Nat Commun. 2022 May 16;13:2700. doi: 10.1038/s41467-022-30328-1 (PMC9110341; doi:10.1038/s41467-022-30328-1)
Supplement: Supplementary file 3 — Description of Additional Supplementary Files [file 41467_2022_30328_MOESM3_ESM.pdf]

Supplementary Movie 1

ATP-loaded NrdR dodecamer (Movie_S1_Dodecamer.mp4). Assembly of dodecamer from three tetramers colored in blue, green and yellow.

Supplementary Movie 2

dATP/ATP-loaded NrdR tetramer bound to DNA (Movie_S2_DNA_bound_Tetramer.mp4). Each monomer of the tetramer colored in blue, red, green and yellow, and dsDNA fragment in gold.

Supplementary Movie 3

dATP/ATP-loaded NrdR octamer (Movie_S3_Octamer). Assembly of octamer from two tetramers colored in blue and green.

Supplementary Movie 4

Morphing of chains A (beige) and B (green) from the ATP-loaded NrdR structure to the dATP/ATP-loaded structure (Movie_S4_morphing_dimer). Morphing path models are only intended to illustrate the differences between the two presented experimental structures.

Supplementary Movie 5

Morphing of chains A (beige), B (green), C (pink) and D (purple) from ATP-loaded dodecamer to dATP/ATP-loaded tetramer states (Movie_S5_morphing_tetramer.mp4). Morphing path models are only intended to illustrate the differences between the two presented experimental structures.
