# Supplementary material for: A nucleotide-sensing oligomerization mechanism that controls NrdR-dependent transcription of ribonucleotide reductases
Source: Nat Commun. 2022 May 16;13:2700. doi: 10.1038/s41467-022-30328-1 (PMC9110341; doi:10.1038/s41467-022-30328-1)
Supplement: Supplementary file 9 — Reporting Summary [file 41467_2022_30328_MOESM9_ESM.pdf]

nature portfolio

Corresponding author(s): Pål Stenmark, Britt-Marie Sjöberg, Derek Logan

Last updated by author(s): Mar 8, 2022

# Reporting Summary

Nature Portfolio wishes to improve the reproducibility of the work that we publish. This form provides structure for consistency and transparency in reporting. For further information on Nature Portfolio policies, see our Editorial Policies and the Editorial Policy Checklist.

## Statistics

For all statistical analyses, confirm that the following items are present in the figure legend, table legend, main text, or Methods section.

| n/a | Confirmed | |
|---|---|---|
| ☐ | ☒ | The exact sample size (*n*) for each experimental group/condition, given as a discrete number and unit of measurement |
| ☐ | ☒ | A statement on whether measurements were taken from distinct samples or whether the same sample was measured repeatedly |
| ☒ | ☐ | The statistical test(s) used AND whether they are one- or two-sided<br>*Only common tests should be described solely by name; describe more complex techniques in the Methods section.* |
| ☒ | ☐ | A description of all covariates tested |
| ☒ | ☐ | A description of any assumptions or corrections, such as tests of normality and adjustment for multiple comparisons |
| ☐ | ☒ | A full description of the statistical parameters including central tendency (e.g. means) or other basic estimates (e.g. regression coefficient) AND variation (e.g. standard deviation) or associated estimates of uncertainty (e.g. confidence intervals) |
| ☒ | ☐ | For null hypothesis testing, the test statistic (e.g. *F*, *t*, *r*) with confidence intervals, effect sizes, degrees of freedom and *P* value noted<br>*Give P values as exact values whenever suitable.* |
| ☒ | ☐ | For Bayesian analysis, information on the choice of priors and Markov chain Monte Carlo settings |
| ☒ | ☐ | For hierarchical and complex designs, identification of the appropriate level for tests and full reporting of outcomes |
| ☒ | ☐ | Estimates of effect sizes (e.g. Cohen's *d*, Pearson's *r*), indicating how they were calculated |

*Our web collection on statistics for biologists contains articles on many of the points above.*

## Software and code

Policy information about availability of computer code

| | |
|---|---|
| Data collection | EPU 2.7 (Thermo Fischer); MST: MO.Control v1.6.1 (Nanotemper Technologies); ITC: MicroCal ITC200 software (Malvern Panalytical); GEMMA: Aerosol Instrument Manager Software Version 5.5.1 (TSI); UNICORN 5.2 control and analysis software (Cytiva) |
| Data analysis | cryoSPARC 3.1, Coot 0.8.5.0, Phenix 1.19, UCSF Chimera version 1.13.1, PyMOL Molecular Graphics System version 2.2.3; MST: MO.Affinity Analysis v2.3 (Nanotemper Technologies); ITC: MicroCal PEAQ-ITC analysis software version 1.1.0.1262 (Malvern Panalytical); GEMMA: Macro IMS Manager software (TSI); SEC: UNICORN 5.2 control and analysis software (Cytiva); Calculation of mean and standard deviation values for all experiments and graphs were made in Microsoft excel. Bioinformatics: sequence alignment using Probcons (v.1.12), logo was created using the Skylign online service, sequence clustered using USEARCH (Edgar 2010), Prokka 1.14.6. |

For manuscripts utilizing custom algorithms or software that are central to the research but not yet described in published literature, software must be made available to editors and reviewers. We strongly encourage code deposition in a community repository (e.g. GitHub). See the Nature Portfolio guidelines for submitting code & software for further information.

## Data

Policy information about availability of data

All manuscripts must include a data availability statement. This statement should provide the following information, where applicable:

- Accession codes, unique identifiers, or web links for publicly available datasets
- A description of any restrictions on data availability
- For clinical datasets or third party data, please ensure that the statement adheres to our policy

Three-dimensional cryo-EM maps generated during this study have been deposited in the Electron Microscopy Data Bank (EMDB) under accession codes EMD-13178 (ATP-loaded NrdR dodecamer), EMD-13179 (dATP/ATP-loaded NrdR tetramer bound to its cognate DNA), and EMD-13182 (dATP/ATP-loaded NrdR


March 2021

octamer). Coordinates of all models have been deposited in the Protein Data Bank (PDB) under accession codes 7P37 (ATP-loaded NrdR dodecamer), 7P3F (dATP/ATP-loaded NrdR tetramer bound to its cognate DNA), and 7P3Q (dATP/ATP-loaded NrdR octamer). The Genome Taxonomy Database is available at (https://gtdb.ecogenomic.org). The RefSeq database is available at (https://www.ncbi.nlm.nih.gov/refseq/).

# Field-specific reporting

Please select the one below that is the best fit for your research. If you are not sure, read the appropriate sections before making your selection.

☒ Life sciences ☐ Behavioural & social sciences ☐ Ecological, evolutionary & environmental sciences

For a reference copy of the document with all sections, see nature.com/documents/nr-reporting-summary-flat.pdf

# Life sciences study design

All studies must disclose on these points even when the disclosure is negative.

**Sample size**

A total of 18,581 cryo-EM movies were recorded, summing up all three samples. The cryo-EM data collection was planned for a total of 3 days of data acquisition, which should, from our general experience, yield enough data for a reconstruction of the structure of the complex. A successful determination of the structure at high resolution proved that the amount of collected data was sufficient. We have used at least 3 different protein preparations (biological replications) for MST, ITC, SEC and GEMMA.

Although it has been estimated that under ideal conditions 12,000 particles should suffice to produce a map that is interpretable at atomic resolution, in practice a larger amount of particle images is often required to achieve near-atomic resolution. The amount of available particles is limited by the concentration of particles on the grid, and the ability to correctly find them in the micrographs and correctly assign their orientation.

Reference: https://www.ncbi.nlm.nih.gov/pmc/articles/PMC3093552/

**Data exclusions**

All movies were analyzed and 55 movies were excluded from further analysis due to low quality and ice artifacts. The first injection in each of the ITC experiments was excluded (standard practice, stated in materials and methods), No other data was excluded.

**Replication**

All cryo-EM replication attempts (protein preparation, cryo-EM grid preparation, data collection and analysis) were successful. The cryo-EM data were collected in a total of 4 independent data collection sessions. Standard deviations were calculated based on ≥3 biological replicates. The information is given in Materials and Methods section and in Extended figures legends.

**Randomization**

No randomization was relevant for this study. Randomization is not relevant for this type of structural biology work. The collected cryo-EM data are random by nature.

**Blinding**

Blinding is not relevant for this type of structural study. A big part of the data processing is based on computer automation and only one type of input data is used (protein complex particles), so there is no risk of bias induced by a lack of blinding.

# Reporting for specific materials, systems and methods

We require information from authors about some types of materials, experimental systems and methods used in many studies. Here, indicate whether each material, system or method listed is relevant to your study. If you are not sure if a list item applies to your research, read the appropriate section before selecting a response.

## Materials & experimental systems

| n/a | Involved in the study |
|---|---|
| ☒ | ☐ Antibodies |
| ☒ | ☐ Eukaryotic cell lines |
| ☒ | ☐ Palaeontology and archaeology |
| ☒ | ☐ Animals and other organisms |
| ☒ | ☐ Human research participants |
| ☒ | ☐ Clinical data |
| ☒ | ☐ Dual use research of concern |

## Methods

| n/a | Involved in the study |
|---|---|
| ☒ | ☐ ChIP-seq |
| ☒ | ☐ Flow cytometry |
| ☒ | ☐ MRI-based neuroimaging |
